# Pupillometry as a Window into Young Children’s Sustained Attention

**DOI:** 10.3390/jintelligence10040107

**Published:** 2022-11-16

**Authors:** Viridiana L. Benitez, Matthew K. Robison

**Affiliations:** 1 Department of Psychology, Arizona State University, Tempe, AZ 85287, USA; 2 Department of Psychology, University of Texas at Arlington, Arlington, TX 76019, USA

**Keywords:** sustained attention, vigilance, pupillometry, children, development

## Abstract

Sustained attention is critical to cognition, social competence, and academic success. Importantly, sustained attention undergoes significant development over the early childhood period. Yet, how sustained attention fluctuates over time on task has not been clearly outlined, particularly in young children. In this study, we provide a first test of whether the pupillary response can be used as an indicator of moment-to-moment sustained attention over time on task in young children. Children aged 5 to 7 years (*N* = 41) completed a psychomotor vigilance task, where they were asked to press a button as fast as possible at the onset of a target stimulus. We measured reaction times over the course of the task, pupil size prior to target onset (baseline pupil size), and pupil size in response to target onset (task-evoked pupil size). The results showed a stereotypical vigilance decrement in children’s response times: as time on task increased, reaction times increased. Critically, children’s task-evoked pupil size decreased over time on task, while no such change was present in baseline pupil size. These results suggest that young children’s waning sustained attention may be linked to a decrease in alertness while overall arousal is maintained. We discuss the importance of leveraging pupillometry to understand the mechanisms of sustained attention over individuals and development.

## 1. Introduction

Sustained attention, the ability to maintain focus on a task for a prolonged period of time, is a hallmark of an intelligent cognitive system. The ability to sustain attention has been found to underlie many important behaviors, including key cognitive processes, such as language, memory, and cognitive flexibility (Benitez et al. 2017; Choudhury and Gorman 2000; Jongman et al. 2015; McQuillan et al. 2021; Yu et al. 2019), skills critical to academic success (Gardner-Neblett et al. 2014; Isbell et al. 2018; Rhoades et al. 2011; West et al. 2021) and social competence (Andrade et al. 2009; Davies et al. 2008). Given the importance of sustained attention, a large body of literature has documented how this ability varies across individuals (Isbell et al. 2018), social and cultural groups (Brandes-Aitken et al. 2019), and clinical populations (Burton et al. 2018; Ebert and Kohnert 2011; Gallardo-Moreno et al. 2020; Huang-Pollock et al. 2020; Rose et al. 2017; Swaab-Barneveld et al. 2000; Tucha et al. 2009; Vivanti et al. 2017).

Of note is how sustained attention abilities change across development (Fortenbaugh et al. 2015). In particular, this ability undergoes significant improvements over the early childhood period (Colombo and Cheatham 2006; Fisher 2019). Although this progression has been well documented using a variety of tasks, this work has largely ignored a key feature of sustained attention—that it can fluctuate from moment to moment over the course of time on a given task (Benitez et al. 2017; Isbell et al. 2018). Understanding the development of sustained attention requires assessing how it is maintained over a prolonged period of time, as well as how the stability of sustained attention changes across individuals and ages (Esterman and Rothlein 2019). In this study, we examine how the pupillary response can provide a measure of the maintenance of sustained attention from moment to moment over time on task in young children.

Measuring the pupillary response has recently become a common tool in psychophysiological research (Laeng et al. 2012; Strauch et al. 2022; Mathôt 2018; Mathôt et al. 2018). Changes in pupil diameter occur automatically, without overt control, making it a method of measurement that can be used across ages and abilities. In adults, changes in pupil size have been linked to alerting, orienting, and executive function networks (Strauch et al. 2022) and have been found to be coupled with arousal, effort, memory, and cognitive control abilities (Just et al. 2003; Laeng et al. 2012). A growing body of work has also linked changes in pupil size to a range of cognitive processes in infants and children, including object processing, learning, memory, predictive processes, and cognitive control (Ackermann et al. 2020; Boersma et al. 1970; Bonmassar et al. 2020; Chatham et al. 2009; Cheng et al. 2019; Eckstein et al. 2017; Hepach and Westermann 2016; Johnson et al. 2014; Karatekin et al. 2007; McGarrigle et al. 2017; Selezneva and Wetzel 2022; Sirois and Brisson 2014; Sirois and Jackson 2012; Zhang et al. 2018; Zhang and Emberson 2020). 

Relevant to understanding sustained attention, changes in pupil size have been proposed as a psychophysiological marker of a neuromechanism connected to alertness and arousal—the locus coeruleus (LC) norepinephrine (NE) system (Aston-Jones and Cohen 2005; Gilzenrat et al. 2010; Rajkowski et al. 1993; Strauch et al. 2022). LC activity can be characterized by the amount of activity at baseline (tonic activity) as well as in response to a task-relevant event (task-evoked activity). During periods of inattentiveness, LC tonic activity is low, LC task-evoked activity is low, and performance on the given task is poor. During periods of focused attention, LC tonic activity is moderate, LC task-evoked activity is high, and performance is high. In contrast, during periods of distractibility, LC tonic activity is high, LC task-evoked activity is low, and performance is poor. As such, the relationship between LC tonic activity and attention is characterized as an inverted-U-shaped relationship. Neural recordings in non-human primates (Joshi et al. 2016; Rajkowski et al. 1993; Varazzani et al. 2015) and neuroimaging studies in human adults (Alnæs et al. 2014; Murphy et al. 2014) have linked LC activity with the pupillary response (see also Eckstein et al. 2017, for a review), indicating that the two are strongly coupled.

Critically, behavioral studies in human adults have linked pupillary responses to moment-to-moment fluctuations in sustained attention (Beatty 1982a, Beatty 1982b; Robison and Unsworth 2019; Unsworth and Robison 2016; Unsworth and Robison 2018; Zhao et al. 2019). Using a psychomotor vigilance task, on each trial, Unsworth and Robison (2016) measured adults’ reaction times to the onset of the target stimulus, task-evoked pupil size changes, indexed as the change in pupil size post target onset, and baseline pupil size, indexed as pupil size prior to the start of a trial. As time on task increased, reaction times increased, demonstrating a vigilance decrement (Dinges and Powell 1985). Importantly, both baseline pupil size and task-evoked pupil size decreased over the course of the task. Further, the pupillary response was linked with different types of self-reported attentional lapses consistent with LC-NE functioning. Compared to high attention states, high arousal but low attention states yielded larger baseline pupil sizes, low arousal and low attention states yielded smaller baseline pupil sizes, and low attention states yielded smaller task-evoked pupil size changes. Thus, baseline pupil size changes seem to be coupled with the LC-NE tonic state, indexing arousal, and task-evoked pupil size changes seem to be coupled with the LC-NE phasic state, indexing alertness or high-intensity attention. In sum, Unsworth and Robison (2016) implicate both arousal and alertness in adults’ moment-to-moment sustained attention over time on task. 

If the pupillary response is linked with fluctuations in sustained attention, then it may be a critical measure for understanding moment-to-moment sustained attention in early childhood. However, no study to date has assessed whether and how the pupillary response is coupled with sustained attention over time on task in young children. A limited set of studies have linked children’s task-evoked pupillary response to mental effort (Boersma et al. 1970; Johnson et al. 2014; Karatekin 2004) and working memory performance (Johnson et al. 2014; Cheng et al. 2019). To our knowledge, however, only two studies have provided preliminary evidence that the task-evoked pupillary response may be linked with moment-to-moment sustained attention in infants (Cheng et al. 2019) and older children (Karatekin et al. 2007).

Cheng et al. (2019) found that, during a working memory task, the task-evoked pupillary response at encoding was larger for 13-month-old infants who were more accurate at test. The authors concluded that high-intensity focused attention at encoding supports the maintenance of visual information in working memory. Using a target detection task, Karatekin et al. (2007) showed that the task-evoked pupil size decreased over time on task in adults and 10-year-old children, but only adults showed a decrease in baseline pupil size. The authors concluded that the decrease in task-evoked pupil size indicated a waning of alertness, while no change in baseline pupil size in children indicated that arousal was maintained throughout the course of the task. These findings hint at a developmental difference in the mechanisms that support sustained attention in older children and adults. However, several questions remain: (1) is the pupillary response coupled with moment-to-moment sustained attention over time on task in young children? and (2) if so, are changes found in both the baseline and the task-evoked pupillary response? Answering these questions can shed light on how arousal (indexed via the baseline pupillary response) and alertness (indexed via the task-evoked pupillary response) supports moment-to-moment sustained attention over time on task in young children.

In the present study, we assessed pupil size changes in 5- to 7-year-old children over time on a psychomotor vigilance task, where children were instructed to press a button when a target stimulus appeared on the screen, with the onset of the target varying randomly across trials (between 1 and 8 s). We measured the reaction time of button presses in response to the onset of the stimulus, as well as the baseline pupillary response (pupil size prior to the onset of the stimulus) and the task-evoked pupillary response (pupil size post onset of the stimulus). If young children’s sustained attention wanes over time, then we should find a vigilance decrement in children’s reaction times. If young children’s pupillary response is coupled with sustained attention, then we should also find that children’s pupillary response shows time-on-task effects. Importantly, by measuring both baseline and task-evoked pupil size changes, we may be able to shed light on the mechanisms behind sustained attention. If both arousal and alertness support the maintenance of sustained attention in young children, then we should find time-on-task effects in both baseline pupil size changes and task-evoked pupil size changes as have been found in adults (Unsworth and Robison 2016).

## 2. Materials and Methods

### 2.1. Participants

Participants were recruited from the local Phoenix metropolitan community through Facebook advertisements, flyers posted on campus and distributed at local preschools, through the Children’s Museum of Phoenix, and by word of mouth. Our goal was to administer the study to as many children as possible over a one-year span, with the intention of collecting at least 82 participants so we could detect age-related correlations of at least 0.30 with 80% power. However, data collection ceased during the COVID-19-related closures in March 2020. We were able to collect half of our target sample (*N* = 41), which meant that our correlation analyses were underpowered.

Our final sample included 41 children (*M* = 6.52 years, *SD* = 0.77, range 5 to 7.9 years). An additional 4 children were excluded due to failure to complete the vigilance task (2) and failure to follow instructions (2). One child was included in the assessment of reaction times but excluded from the pupillary analyses due to an error in recording the eye data. The final sample was composed of 22 girls, 19 boys, 15 children whose parents indicated they were exposed to a language other than English, and 26 indicating no exposure to a second language. Our sample was racially and ethnically diverse. The racial/ethnic breakdown, as reported by the parent, was the following: White (17), Hispanic/Latino (7), Black/African American (6), Asian (4), American Indian or Alaskan Native (1), two or more racial categories (5), and other (1). Parents also reported on their highest level of education, with 33 holding a Bachelor’s degree or higher, 7 having attended some college or holding an Associate’s/technical degree, and 1 with less than a high school degree. Additionally, 3 children were reported to have a developmental impairment. We opted to include these three children to be most data-inclusive, as they were able to follow the instructions to complete the task (results remain qualitatively the same if these children are excluded).

The children and their families were invited to the Learning & Development Lab at Arizona State University (ASU) as part of a larger study on individual differences in cognitive abilities. During the session, in addition to the psychomotor vigilance task, children completed four tasks not reported here: a change-detection working memory task, the NIH Toolbox Cognition Battery flanker task (Zelazo et al. 2013), and a cross-situational word-learning task (Benitez et al. 2020). The psychomotor vigilance task was always completed first. The children received a book, t-shirt, or USD 10 in cash for participating in the study. Parental consent was obtained for all participants in accordance with the ASU Institutional Review Board.

### 2.2. Task Design and Procedure

**Figure 1:**
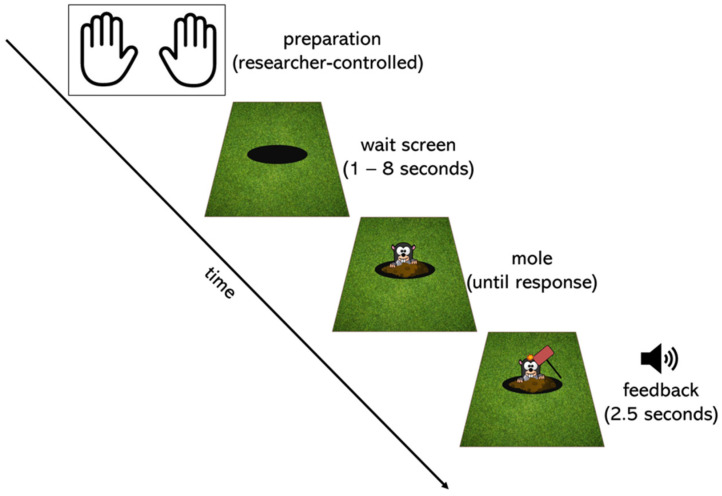
Graphical depiction of the task sequence. Each trial was initiated by the experimenter, ensuring that the child was in position (preparation). A black circle (a hole) on a green background (grass) then appeared for a random time wait interval ranging from 1 to 8 s (wait screen). After this interval, a mole appeared, and the child’s task was to press the button on the response box as quickly as possible (mole). When they did so, a hammer came down on the mole, and a laughing sound played for 1.5 s (feedback). Stars on the right-hand side of the screen told participants how many trials they had completed in the block. The preparation screen for the next trial then began.

Children completed a modified version of the psychomotor vigilance task (Dinges and Powell 1985). See Figure 1 for a graphical depiction of the task sequence. The task was programmed in Python. Each trial began with a screen that presented a black circle (a hole) surrounded by a green background (grass). After a random wait period ranging from 1 to 8 s, a picture of a mole appeared. Children were instructed to “catch” the mole by pressing a response button as quickly as possible when they noticed the mole. When the child pressed the button, the task provided auditory and visual feedback: a hammer came down on the mole’s head, and a laughing sound played. In addition, stars appeared on the right-hand side of the screen, indicating how many trials they had completed successfully in that block. The task included a practice block as well as at least 3 testing blocks of 10 trials each.

The task was completed inside a single-walled sound-attenuated booth, with children seated comfortably at a table to which a chinrest was mounted. The table was in front of a height-adjustable ViewSonic LCD monitor (1920 × 1080), approximately 18 inches from eye level, with the main stimulus being 17 degrees of visual angle in size. The lighting in the booth was set on the same brightness setting (medium) for all children. The brightness at eye level was approximately 285 lux during the task. The children were told that they were going to play a game on the computer called whack-a-mole, during which they would have to catch a mole. At the start of the session, the children were instructed to place their chins on the chinrest. The chinrest was surrounded by paper-mâché “rocks” in a way that did not interfere with the eye-tracker, and the participants were instructed that they must place their chins on the chinrest during the game so they could “hide” from the mole. A researcher stood behind the child during the entire task so they could instruct and monitor the child. A second researcher sat at the control computer outside the booth running the task. The second researcher could hear and see the child and the researcher via a camera. The researcher inside the booth was instructed to signal to the researcher outside the booth to initiate each phase and trial of the task.

Once the child was seated and in the chinrest, a 5-point child-friendly calibration procedure was initiated, where a rubber duck was displayed (together with a ringing sound) at the center and 4 corners of the monitor. Children were instructed to look at the duck. The researcher outside the booth pressed a key when the child was looking at the stimulus. The calibration procedure was repeated a second time if needed.

After calibration, children completed several practice trials in order to become oriented to the task. Each trial (for practice and test trials) started with a screen instructing participants to place one hand on the button response and the other hand next to the response box placed on the table in front of them (see “preparation” in Figure 1). When the research assistant ensured the child’s hands were in position and the child was attentive to the screen, they signaled to the outside researcher to initiate the trial. 

During the practice trials, the researcher provided feedback to children to try and press the button as fast as possible with the same hand, to maintain their chin on the chinrest, and to place their hands at the start position at the start of each trial. If the child failed to press the button after 5 s, the trial would end, and a red X would appear—these trials would be counted as incorrect. Children were presented with 5 practice trials. If a child was incorrect on 1 or more practice trials, they would restart the practice phase. Only after children completed all 5 practice trials correctly did children move on to the test trials. In this way, we emphasized to children to make as few errors as possible during the task.

The test trials were presented in blocks of 10 trials each. All children were asked to complete at least three blocks of 10 trials correctly. Children were encouraged to complete more trials, up to 6 blocks. However, these additional trials were not analyzed since children varied with respect to how many trials they completed beyond the 3 blocks required. Thus, only the first 30 correct test trials were analyzed for all children. Once children completed a block, they were given a sticker to place on a sticker sheet, and the next block started. If children were incorrect on a trial, that trial was discarded, and a new trial was presented to replace it. This ensured that children completed 10 correct trials for each block. No RT or eye data were collected on incorrect trials (these composed only 0.04% of the total data). The entire task lasted about 6 min. After completion of all the tasks, parents completed a demographic questionnaire for their child. The entire session lasted approximately 45 min.

### 2.3. Pupillometry

While children completed the psychomotor vigilance task, their gaze position and pupil diameter were recorded from both eyes via a Tobii X3-120 eye tracker mounted to the bottom of the monitor. We used the pupil diameter of the right eye for our analyses consistent with prior research (Unsworth and Robison 2016; left and right pupil sizes were highly correlated, *r* = 0.82, *p* = < .001; the results were qualitatively the same when the left eye was used). Only valid pupil data were included in the analyses (invalid pupil data due to missing data or blinks were excluded). From the pupil data, we extracted two measures. First, we measured the baseline pupil diameter by averaging the pupil values over the first second of the wait screen. We picked the first second of the wait screen given that the length of the wait screen varied randomly between 1 and 8 s. Second, we computed a task-evoked pupillary response by examining changes in pupil diameter in response to the appearance of the mole. To do so, for each trial, we calculated the pupil size change by subtracting the average pupil size over the 2000 ms time window post-target onset from the average pupil size over the 500 ms time window before target onset, consistent with prior research (Unsworth and Robison 2016).

### 2.4. Data Analysis

We used R for all of our analyses. To aggregate and transform data, we used *tidyverse* (Wickham 2017) and *data.table* (Dowle and Srinivasan 2018) packages; to estimate linear mixed effects models, we used the *lmerTest* package (Kuznetsova et al. 2017); to plot figures, we used the *ggplot* (Wickham 2016) and *cowplot* (Wilke 2020) packages. The data and R markdown file are openly accessible at https://osf.io/q5fzh/.

## 3. Results

### 3.1. Reaction Time (RT)

**Figure 2:**
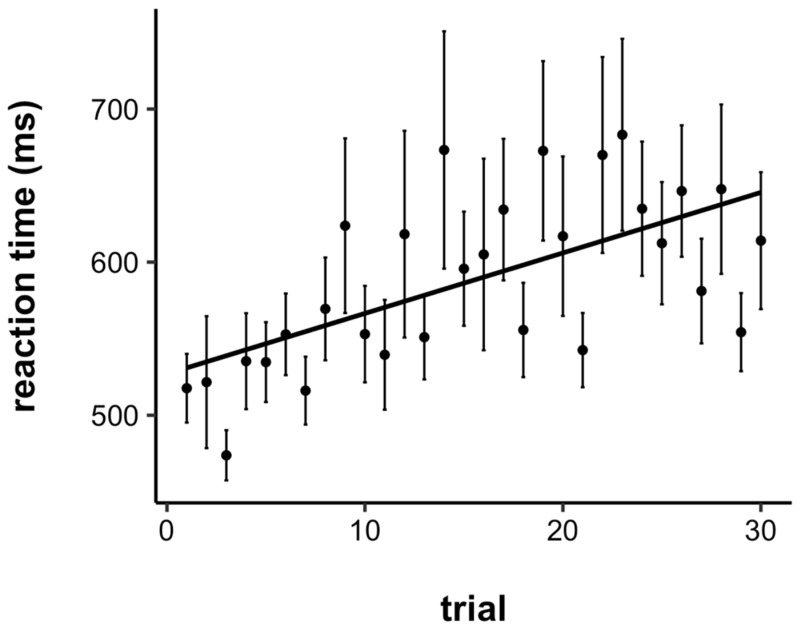
Average reaction time by trial. Children showed a significant vigilance decrement, with reaction times slowing across the course of the task. Error bars represent +/− one standard error.

The RTs were trimmed by removing any RT faster than 200 ms (anticipations) and longer than 3000 ms. This process only excluded 0.04% of RTs across all 41 participants. The average RT by trial is plotted in Figure 2. To statistically examine task performance as a function of time-on-task, RTs were submitted to a linear mixed effects model with a fixed effect of trial number and a random intercept for each participant (a model with the additional random slope for each participant led to a singular fit). Overall, there was a significant slowing of RTs across trials (*b* = 4.02, *SE* = 0.79, *p* < .001). Therefore, children did indeed show a vigilance decrement, even on a rather short task (~6 min).

### 3.2. Pupillometry

**Figure 3:**
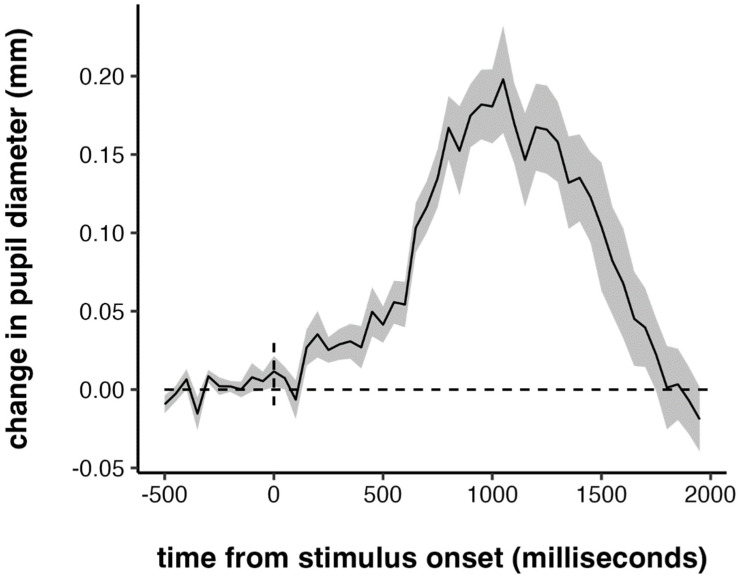
Grand-averaged task-evoked pupillary response. Shaded error bar represents +/− one standard error.

Our next set of analyses focused on pupillary measures. First, we examined the time course of the task-evoked pupillary response, averaged across all trials and all participants, from 500 ms prior to target onset to 2000 ms post-target onset. The time course of the pupillary response is plotted in Figure 3. Children’s tasked-evoked pupillary response was quite similar to that observed in adults with other psychomotor vigilance tasks in shape, magnitude, and latency (Massar et al. 2016; Massar et al. 2019; Robison 2018; Unsworth and Robison 2016; Unsworth and Robison 2017). The peak of the response happened around 900–1000 ms, which lags behind the peak of the task-evoked pupillary response in adults by about 200 ms. This is consistent with the lag in RTs between adults and children: the average RTs in children were also about 200 ms slower than what is typically seen in adults.

**Figure 4:**
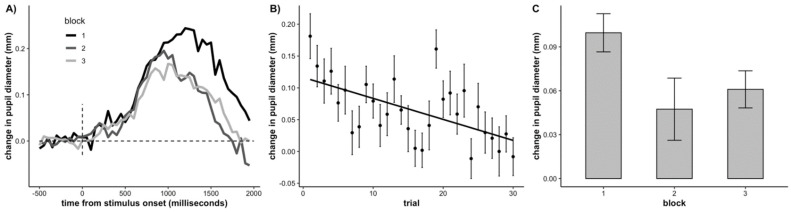
(**A**) Waveforms of the task-evoked pupillary response by block, (**B**) Average change in pupil size at target onset for each trial, (**C**) Average change in pupil size at target onset across each block. Error bars represent +/− one standard error.

Next, we examined time-on-task effects on the task-evoked pupillary response. To do so, we computed a change in pupil size score trial-by-trial by first averaging the pupil size prior to target onset (time window: −500 ms to 0 ms) and subtracting this average from the average pupil size post target onset (time window: 0 to 2000 ms). We submitted the change in pupil size values to a linear mixed effects model with a fixed effect of trial (continuous, mean-centered) and participant as a random effect. Both the intercept and slope were allowed to vary across participants. There was a significant effect of trial, demonstrating that change in pupil size significantly decreased across trials (*b* = −0.0032, *SE* = 0.0007, *p* = < .001), replicating the typical pattern observed in adults (Unsworth and Robison 2016; the results were the same when we time-locked the task-evoked pupillary response to the button press). For visualization, we have plotted the time course of the average task-evoked response in each block of trials in Figure 4A, the average change in pupil size across trials in Figure 4B, and the average change in pupil size across blocks in Figure 4C.

**Figure 5:**
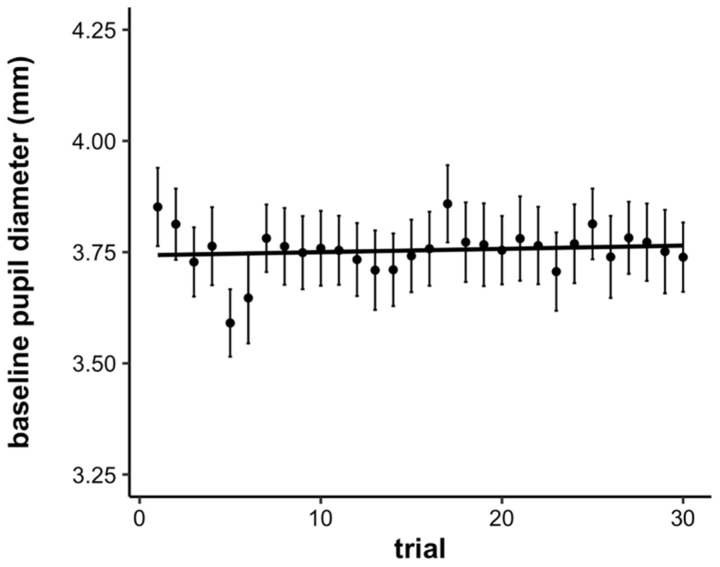
Average baseline pupil diameter by trial. Error bars represent +/− one standard error.

We additionally examined time-on-task effects on the baseline pupillary response. To examine the baseline pupillary response statistically, we computed the average pupil diameter during the first second of the waiting period for each trial. Then, we submitted these values to a linear mixed effects model with a fixed effect of trial (continuous, mean-centered) and a random effect of participant. Both the intercept and slope were allowed to vary across participants. There was no significant effect of trial on the baseline pupil diameter, showing that overall, baseline pupil diameter did not significantly change with time-on-task (*b* = 0.001, *SE* = 0.001, *p* = 0.47; see Figure 5).

### 3.3. Age Related Differences

We next examined the links between age and our key measures as exploratory analyses. These should be interpreted with caution, given that our sample was underpowered to detect correlations; we report confidence intervals to aid in interpretation. To estimate individual vigilance decrements, we specified separate linear models for each participant and used participants’ slope as an indicator of the magnitude of the vigilance decrement. 

**Figure 6:**
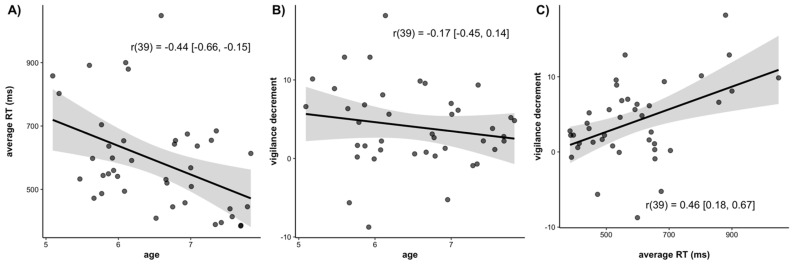
Scatterplots of the correlations between (**A**) Age and average reaction time, (**B**) Age and the magnitude of the vigilance decrement, and (**C**) Average reaction time and magnitude of the vigilance decrement. Lines of best fit are drawn through the data points with associated standard errors. Correlation values are listed with the 95% confidence interval around the estimate.

In Figure 6, we have plotted correlations among average RT, the magnitude of the vigilance decrement, and age. There was a significant negative correlation between age and average RT (*r*(39) = −0.44, 95% CI = [−0.66, −0.15], *p* = 0.004; Figure 6A). Older children had faster RTs than younger children. There was a negative but non-significant correlation between the magnitude of the vigilance decrement and age (*r*(39) = −0.17, 95% CI = [−0.45, 0.14], *p* = 0.28; Figure 6B). This indicates a trend for older children to have a smaller vigilance decrement than younger children. There was a significant positive correlation between average RT and the magnitude of the vigilance decrement (*r*(39) = 0.46, 95% CI = [0.18, 0.67], *p* < 0.001; Figure 6C), such that children who were slower overall also tended to show larger vigilance decrements.

**Figure 7:**
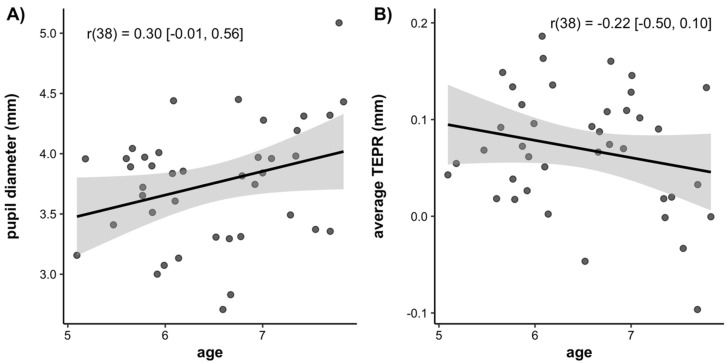
Scatterplots of the correlations between (**A**) Age and average baseline pupil diameter, (**B**) Age and average task-evoked pupillary response. Lines of best fit are drawn through the data points with associated standard errors. Correlation values are listed with the 95% confidence interval around the estimate.

Next, we examined the correlations between average baseline pupil diameter, average task-evoked pupillary response (indexed as the average change in pupil size from pre- to post-target onset), and age. These correlations are plotted in Figure 7. There was a trend for a positive correlation between age and baseline pupil diameter, but this correlation did not reach significance (*r*(38) = 0.30, 95% CI = [−0.01, 0.56], *p* = 0.06). This trend suggests that the baseline pupil diameter may increase over age. There was also a trend for a negative correlation between age and the average task-evoked pupillary response, suggesting that it decreases over age. However, this correlation was not significant (*r*(38) = −0.22, 95% CI = [−0.50, 0.10], *p* = 0.17).

### 3.4. Fixations 

**Figure 8:**
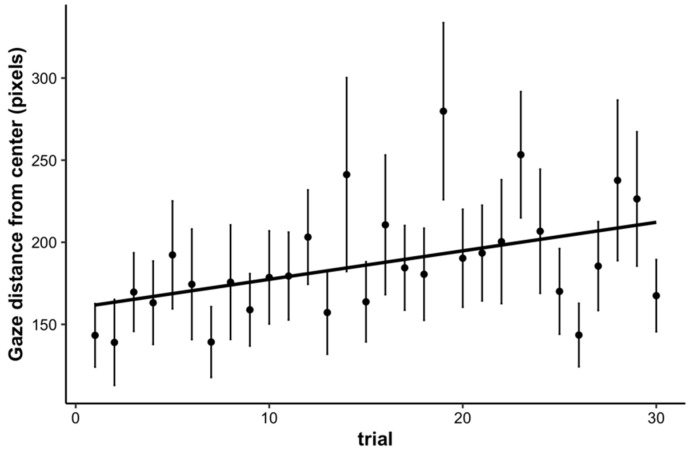
Average distance from the center of the screen of children’s fixations (in pixels) across trials. Children showed a significant increase in distance from center over the course of the task. Error bars represent +/− one standard error.

Finally, as an exploratory analysis, we examined children’s fixations 500 ms prior to target onset to 500 ms post target onset to assess how much their gaze deviated from the center of the screen where the main stimulus was positioned (we thank an anonymous reviewer for this suggestion). For each trial, we computed the average deviation (in pixels) of children’s fixations in Euclidean distance. We submitted the average distance from center to a linear mixed effects model with trial as a fixed effect and a random intercept for participant. The results showed a significant increase in average distance from center over the course of the task (*b* = 1.54, *SE* = 0.49, *p* = 0.002). These results suggest that as time on task increased, children’s eyes began to wander away from the main stimulus, indicating a waning of sustained attention (see Figure 8). 

As a final analysis, we examined if the effect of trial on the task-evoked pupillary response was still present if we took into consideration children’s wandering eyes. We conducted a linear mixed effects model predicting the task-evoked pupillary response, with trial and average gaze distance from center as fixed effects and a random intercept for participant. Consistent with our initial results, trial significantly predicted the task-evoked pupillary response, demonstrating a decrease in the task-evoked pupillary response over time on task (*b* = −0.0033, *SE* = 0.0008, *p* = < .001). However, the average gaze distance from center did not significantly predict the task-evoked pupillary response (*b* = −0.000028, *SE* = 0.000046, *p* = 0.54). This suggests that children’s wandering eyes did not account for the change in the task-evoked pupillary response over time on task. 

## 4. Discussion

In this study, we examined if the pupillary response is linked with the maintenance of sustained attention over time on task in young children. What we found is strong evidence that the pupillary response is coupled with children’s sustained attention performance. In a psychomotor vigilance task, children displayed a stereotypical vigilance decrement in reaction time scores; as time on task increased, their reaction times increased. Importantly, although the baseline pupillary response did not change over time on task, the task-evoked pupillary response did: the pupillary response to the onset of the stimulus decreased over the course of the task. Our results provide strong evidence for a link between the task-evoked pupillary response and waning sustained attention over time on tasks in young children.

The decrease in the task-evoked pupillary response over time on task is consistent with prior studies in adults showing that the task-evoked pupillary response is largest in moments of focused attention states, compared with off-task attention states (Unsworth and Robison 2016) and with findings that the task-evoked pupillary response decreases as vigilance decreases in both adults and older children (Karatekin et al. 2007). Critically, our results yield some insights into the mechanisms that may underlie the maintenance of sustained attention in young children. In particular, if the pupillary response is a psychophysiological marker of LC-NE activity, as previous studies have proposed (Alnæs et al. 2014; Aston-Jones and Cohen 2005; Gilzenrat et al. 2010; Joshi et al. 2016; Murphy et al. 2014; Rajkowski et al. 1993; Varazzani et al. 2015), then the current results show that over the course of the task, as children’s performance deteriorated, their task-evoked pupillary response decreased, suggesting that alertness waned. However, the lack of change in baseline pupil diameter indicates that arousal was maintained over the course of the task. These findings are in line with the only other study we know of that has tested the link between older children’s sustained attention and both baseline and task-evoked pupillary responses (Karatekin et al. 2007). Together, these results point to the possibility that the mechanism behind maintaining sustained attention in children, at least under the conditions tested here, may be more heavily dependent on the ability to maintain alertness than the ability to maintain optimal levels of arousal.

It is important to note that the mechanisms recruited for sustaining attention over time on a task may depend on the particulars of the task itself. If baseline pupil size changes are an index of LC tonic activity and arousal, it is quite possible that we may see changes in this measure in young children if the task was longer, more difficult, or if the reward for performance was manipulated. Indeed, children were continuously motivated to perform well on the task by being awarded a star on the screen for every trial completed and stickers for every block completed. Children were also provided with auditory feedback (a laughing mole) with every successful response. Given that previous research has found that reward improves sustained attention in adults (Massar et al. 2016), the stars, stickers, and laughing mole may have allowed children to maintain optimal levels of arousal throughout the task. Further, although children were allowed to re-set before the start of the next test trial, we cannot discard the possibility that feedback from the prior trial could have impacted the baseline pupillary response on the next trial. In future studies, it will be important to manipulate the time on task, difficulty, and presence of reward to better understand how these influence baseline and task-evoked measures of the pupillary response under different conditions in young children.

We also have to acknowledge that the discrepancy in the time-on-task effects between the task-evoked pupillary response and the baseline pupillary response may not necessarily reflect different mechanisms of attention and instead may reflect that these two pupillary markers are differentially sensitive to the demands of the task. Previous research has consistently shown that the task-evoked pupillary response decreases over time on task (Beatty 1982a, Beatty 1982b; Robison and Unsworth 2019; Unsworth and Robison 2016; Unsworth and Robison 2018; Zhao et al. 2019). However, time-on-task effects on the baseline pupillary response have been less consistent: in adults, although several studies have shown effects of time-on-task on the baseline pupillary response (Kristjansson et al. 2009; Murphy et al. 2011; Unsworth and Robison 2016; van den Brink et al. 2016), others have shown no effects (Martin et al. 2022; Beatty 1982b). Further, increases and decreases in baseline pupil size changes may each be related to different types of waning attention (Unsworth and Robison 2016). Future work is needed to better understand how the baseline pupillary response is coupled with sustained attention performance, not just in adults but also in young children. 

We additionally explored how fixations change over the course of the task, and we found that as time on task increased, the distance of children’s fixations from the center (where the main stimulus was presented) increased. This result shows that children’s eyes started to wander away from the target stimulus over the course of the task and is consistent with children’s RT performance and the task-evoked pupillary response, indicating that children’s sustained attention waned. However, when accounting for gaze distance from center, we still found a significant effect of trial on the task-evoked pupillary response, demonstrating that children’s wandering eyes did not account for the changes in the task-evoked pupillary response over time on task. 

Nonetheless, we do have to acknowledge that because eye movements affect the accuracy of pupil measurement, children’s eye movements may have impacted the measurement of the task-evoked pupillary response (Hayes and Petrov 2016; Gagl et al. 2011; Brisson et al. 2013). This issue is not unique to our study; it is difficult for even adults to maintain fixation consistently over the course of a long task. There is yet no strong consensus on best practices for how to account for eye movements when measuring the pupillary response (Mathôt et al. 2018). We suggest here that developing such best practices should take into consideration conditions for testing young children. 

Although it is clear that sustained attention undergoes dramatic improvements over development, and large increases occur over early childhood (Colombo and Cheatham 2006; Fisher 2019; Fortenbaugh et al. 2015), what is less clear are the mechanisms underlying these changes. What our study demonstrates is that we can leverage pupillometry to understand the psychophysiological drivers of sustained attention, even in a very young sample. Demonstrating this link is the first step in understanding what factors contribute to moment-to-moment shifts in sustained attention. One next step is to understand how these mechanisms change across development. Although our study included a relatively small age span, we found several trends in our correlations between age and our measures even in this short window of development: performance on the vigilance task was better in older children than younger children, the baseline pupillary response was larger in older children than younger children, and the task-evoked pupillary response was smaller in older children than younger children. In future studies with a larger age span, a larger sample size, and an adult comparison, we will be able to measure the pupillary response over time on task across ages to gain insights into how mechanisms of sustained attention change over development. 

Finally, the pupillary response may be harnessed to understand individual differences in cognitive abilities. Several studies have linked adults’ individual pupillary response in sustained attention tasks with their attentional control, working memory capacity, long-term memory, processing speed, and intelligence (Ahern and Beatty 1979; Coors et al. 2022; Robison and Brewer 2022; Tsukahara et al. 2016; Tsukahara and Engle 2021; but see Coors et al. 2022 and Robison et al. 2022 for failures to replicate the link between intelligence and resting pupil size). A limited set of studies have examined individual differences in the ability to maintain attention over time in early childhood, suggesting that this ability is linked with individual differences in other cognitive abilities. In a sample of 3- to 6-year-old children, Benitez et al. (2017) demonstrated that children who were able to maintain their attention selectively to a specific dimension over time were also children who were able to flexibly switch between dimensions in the Dimensional Change Card Sort (DCCS) Task, a measure of cognitive flexibility (Frye et al. 1995). In a different study, Isbell et al. (2018) demonstrated that 3- to 6-year-old children who displayed higher attentional fluctuations, indexed via higher variability in reaction times in a Go/No go task, were also children who performed worse not only on measures of cognitive flexibility (the DCCS task) but also on measures of academic performance (tests of math and reading readiness). If the pupillary response is linked with young children’s sustained attention, as our findings indicate, then it may also predict different domains of cognitive abilities as well as academic performance. This is an important avenue to explore in future research.

## 5. Conclusions

Sustained attention can fluctuate from moment to moment over time on a given task. Here, we provide the first evidence that the pupillary response is coupled with sustained attention over time on task in 5- to 7-year-olds. Specifically, as young children’s reaction times increased over time on task, their task-evoked pupillary response decreased, while their baseline pupillary response did not change. These results suggest that young children’s waning sustained attention may be linked to waning alertness while overall arousal was maintained. Our study provides strong evidence that pupillometry can be harnessed to understand the mechanisms of sustained attention in young children and points to future research needed to understand these mechanisms across individuals and development.

## Data Availability

All data reported in this manuscript are openly accessible at https://osf.io/q5fzh/.
